# The Genus *Haplophyllum* Juss.: Phytochemistry and Bioactivities—A Review

**DOI:** 10.3390/molecules26154664

**Published:** 2021-07-31

**Authors:** Majid Mohammadhosseini, Alessandro Venditti, Claudio Frezza, Mauro Serafini, Armandodoriano Bianco, Behnam Mahdavi

**Affiliations:** 1 Department of Chemistry, College of Basic Sciences, Shahrood Branch, Islamic Azad University, Shahrood 3616713455, Iran; 2 Dipartimento di Chimica, Università di Roma “La Sapienza”, Piazzale Aldo Moro 5, 00185 Rome, Italy; alessandro.venditti@gmail.com (A.V.); armandodoriano.bianco@fondazione.uniroma1.it (A.B.); 3 Dipartimento di Biologia Ambientale, Università di Roma “La Sapienza”, Piazzale Aldo Moro 5, 00185 Rome, Italy; mauro.serafini@uniroma1.it; 4 Department of Chemistry, Faculty of Science, Hakim Sabzevari University, Sabzevar 9617976487, Iran; behnammahdavi@yahoo.com

**Keywords:** *Haplophyllum* Juss. genus, Rutaceae, phytochemistry, chemotaxonomy, ethnobotany, bioactivities

## Abstract

Herein, a comprehensive review is given focusing on the chemical profiles of the essential oils (EOs), non-volatile compounds, ethnobotany, and biological activities of different *Haplophyllum* (Rutaceae family) species. To gather the relevant data, all the scientific databases, including Scopus, ISI-WOS (Institute of Scientific Information-Web of Science), and PubMed and highly esteemed publishers such as Elsevier, Springer, Taylor and Francis, etc., were systematically retrieved and reviewed. A wide array of valuable groups of natural compounds, e.g., terpenoids, coumarins, alkaloids, lignans, flavonoids, and organic acids have been isolated and subsequently characterized in different organic extracts of a number of *Haplophyllum* species. In addition, some remarkable antimicrobial, antifungal, anti-inflammatory, anticancer, cytotoxic, antileishmanial, and antialgal effects as well as promising remedial therapeutic properties have been well-documented for some species of the genus *Haplophyllum*.

## 1. Introduction

It is evident that herbal and medicinal plants play a vital role on the life of human beings and have unique compartment in their lifestyles. Over the past few decades, a large number of scientific investigations have been carried out on a wide spectrum of herbal plants and these attempts have led to the isolation of a large number of valuable natural compounds in different plant species [1,2]. In reality, medicinal plants are used in different scientific disciplines, from food industries to the fragrance and cosmetics domain, to different medicinal and pharmaceutical approaches [3,4].

*Haplophyllum* Juss. is a genus of plant species belonging to the Rutaceae family and comprises 160 species of which only two are accepted, i.e., *Haplophyllum dauricum* (L.) G. Don and *Haplophyllum suaveolens* Ledeb., whereas fifty species are considered to be synonyms and one hundred and eight are unresolved names [5].

The etymology of the name derives from the union of two Greek words, απλοũς (haplous), meaning simple, and φύλλον (phýllon), meaning leaf in the sense of a simple leaf. These terms refer to the fact that the species belonging to this genus are characterized by non-composite leaves.

**Figure 1:**
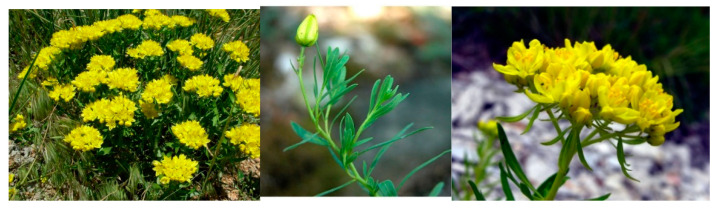
The photographs of *Haplophyllum suaveolens* Ledeb.

From a botanical standpoint, these species appear mainly as perennial herbs even if low shrubs also exist. They present cymose and bracteate inflorescences, with petals being variably colored from light white to bright yellow. They have ten stamens and have free filaments which are widely expanded below and are pubescent on the inner surface (Figure 1) [6].

The distribution area of this genus is quite wide, ranging from Morocco and Spain to China and passing through Romania, Somalia, Turkey, Iran, and Central Asia [6]. Additionally, many relevant species are endemic and some even occur in small, unlinked populations. In particular, the latter characteristics concern the Iranian and Central Asian species, and, for this reason, the genus is locally and partially considered to be very susceptible to extinction [7].

In the present review article, we aimed to cover and discuss the available phytochemical knowledge involving the composition of the chemical profiles of *Haplophyllum*’s essential oils (EOs) as well as the characterized non-volatile compounds and their relevant biological activities. This work represents an updating, an extension, as well as a partial modification of the work by Prieto et al. [8] on the phytochemistry and bioactivities of the same genus. To collect the corresponding data, Scopus (date of access: 20 January 2021 and revisited on 06 June 2021), PubMed (date of access: 10 January 2021 and revisited on 05 June 2021), ISI-WOS (date of access: 21 January 2021 and revisited on 05 June 2021), and a number of published reports dealing with different aforementioned aspects were carefully studied. The keywords used for this research were *Haplophyllum*, phytochemistry, ethnobotany, ethnopharmacology, pharmacology, and biological activities, in combination between *Haplophyllum* and the rest of the mentioned keywords, one by one. The systematic research was also conducted considering all the accepted or unresolved names of *Haplophyllum* species, as reported in www.theplantlist.org, accessed on 24 June 2021 [5], alone or in combination with the previous terms, one by one. All the *Haplophyllum* species, now taxonomically considered to be synonyms of other species, as reported in www.theplantlist.org, accessed on 24 June 2021 [5], were not taken into consideration for this review. In any case, all the existing works, abiding by these rules, were inserted in spite of the years or types of publications.

## 2. Phytochemistry

The *Haplophyllum* species have been studied for their phytochemical constituents that regard both the EOs and the polar fraction metabolites.

### 2.1. Essential Oils of Haplophyllum Species

EOs could be defined as hydrophobic liquid mixtures usually having a lower density of water and comprising versatile natural compounds that are separated using different approaches, e.g., expression, cold press, water-distilled extraction, steam distillation, and numerous microwave-based techniques [9,10,11]. Within the past few decades, EOs have gained much attention due to their widespread uses in a variety of phytochemical, biological, medicinal, pharmaceutical, and food disciplines as well as in the flavour and fragrance industry [12,13]. In fact, a large number of reports could be found in the literature highlighting the remarkable potential use of EOs for a wide spectrum of applications [14,15]. Similar to many other herbal genera, *Haplophyllum* species are considered as valuable sources of secondary metabolites such as EO components. According to the literature, a large number of reports have argued the chemical profiles of the EOs obtained from different organs of *Haplophyllum* species. Table 1 displays the main compounds identified in the EOs of different Haplophyllum species.

Table 2 shows the distribution of the main volatile compounds in the *Haplophyllum* spp. essential oils.

As it can be seen from Table 2 and Table 3, the literature data concerning the chemical profiles of the EOs of this valuable medicinal genus are abundant, in particular about its most important species, i.e., *H. tuberculatum* (Forssk.) A. Juss. From a general survey of these data, it could be clearly observed that the characterized chemical profiles of this species differ widely from one another. Yet, these profiles were mainly seen to be characterized by the presence of monoterpene hydrocarbons (MH), oxygenated monoterpenes (OM), and non-terpene hydrocarbons (NH). Other reported classes are also sesquiterpene hydrocarbons (SH) and oxygenated sesquiterpenes (OM), even if with minor frequency. This same pattern was also reported in several other species such as two *Hyptis* species (Lamiaceae family) [40], several *Hypericum* species (Hypericaceae family) [41] and *Helichrysum* species (Asteraceae family) [42]. Not all the compounds were reported in all the species. Nevertheless, the most reported compounds were β-caryophyllene and β-pinene [17,18,19,20,21,23,25,26,28,31,32,39], whereas several compounds were identified only in single species.

For what concerns the phytochemical profiles of *H. tuberculatum*, in some reports, the major compounds were limonene, α-pinene, β-pinene, α-phellandrene, β-phellandrene, myrcene, δ-3-carene, β-ocimene, α-terpinene [37], and β- and γ-terpinene [30,31,32,33,37], whereas, in others, the major components were linalool, linalyl acetate, 1,8-cineole, 4-terpineol [37], *trans*-*p*-menth-2-en-1-ol, *cis*- and *trans*-*p*-menth-2-en-1-ol, piperitone, and *cis*- and *trans*-piperitol [29,31,34,36,37,38]. As shown in Table 1, for what concerns the volatile fractions and oils from *H. myrtifolium* specimens, monoterpene hydrocarbons [23] or non-terpene hydrocarbons were the prevailing groups of natural compounds [23,24]. Monoterpene hydrocarbons and oxygenated monoterpenes were the main class of constituting compounds of *H. robustum* Bunge [26,27,28]. On the other hand, some sporadic reports dealt with the isolation and identification of the volatile essences of other species of the genus *Haplophyllum*. In accordance with these reports, monoterpene hydrocarbons were the most abundant compounds in *H. glaberrimum*, *H. virgatum*, *H. laeviusculum*, and *H. buhsei* [17,19,39], whereas, for *H. virgatum*, *H. buxbaumii*, and *H. megalanthum*, non-terpene hydrocarbons were found in the highest quantities [18,21,22]. *H. acutifolium* oil consisted mainly of sesquiterpene hydrocarbons [16]. It is also interesting to note that the total amounts of monoterpene hydrocarbons and oxygenated sesquiterpenes in the *H. furfuraceum* oil were approximately the same [18]. Lastly, by using the headspace solid phase microextraction (HS-SPME) approach, volatile fractions from the flowers and stems of *H. perforatum* Kar & Kir. were observed to be mainly composed of monoterpene hydrocarbons, whereas that of the leaves contained higher quantities of sesquiterpene hydrocarbons [25].

### 2.2. Polar Fraction Metabolites of Haplophyllumn Species

Regarding the non-volatile fraction metabolites, *Haplophyllum* species biosynthesize compounds belonging to the class of terpenoids, saponins, alkaloids, coumarins, lignans, flavonoids, and organic acids (Table 3 and Figure 2, Figure 3, Figure 4, Figure 5, Figure 6, Figure 7, Figure 8, Figure 9, Figure 10, Figure 11, Figure 12, Figure 13 and Figure 14).

As it can be seen from Table 3, not all the *Haplophyllum* species were studied for their non-volatile components. Surely, alkaloids, coumarins, and lignans represent the most represented classes of natural compounds in this genus, having been reported in most of them, often together, even if some exceptions are present (i.e., *H. canaliculatum*, *H. kowalenskyi* and *H. tenue*, where only alkaloids were identified [57,77] and *H. dshungaricum*, where only coumarins were identified) [67]. In addition, only for the species *H. alberti-regelii*, one compound was identified [49], whilst for all the others, at least two compounds were identified, even if they belonged to the same phytochemical class. For some species and/or exemplars, the exact compounds were not specified since only a phytochemical screening was performed such as for *H. boissierianum*, *H. glaberrimum*, *H. pedicellatum*, and *H. tuberculatum* from Iran and *H. robustum* and *H. suaveolens* from Serbia [50,72]. The extraction solvents are well-known as well as the analysis methods. Of course, their choice depends on the kind of compounds that need to be extracted from the *Haplophyllum* species. Ethanol proved to be a very effective solvent to extract different classes of compounds, both polar and non-polar, whilst dichloromethane, methanol, *n*-hexane, petroleum ether, chloroform, and ethyl acetate were perfect for extracting compounds such as alkaloids, lignans, and coumarins. For what concerns the studied organs, these are quite general, too, with a prevalence of aboveground organs. Indeed, for what concerns the collection areas of the studied species, the general knowledge of the *Haplophyllum* genus geographical distribution is respected since the majority of them were collected in Asia.

Table 4 displays the distribution of the phytochemical compounds within the *Haplophyllum* genus.

As it can be seen from Table 3, the distribution of the compounds is not equable in all the *Haplophyllum* species. Alkaloids have been reported as the most representative compounds in the genus, and they are also of the utmost importance from a chemosystematic standpoint [114]. Skimmianine is the most reported compound of this class in the genus, followed by γ-fagarine [44,45,46,49,51,52,53,54,57,58,66,73,75,77,78,80,81,82,85,86,87,89,90,95,97,98,101,106,108,111]. Coumarins were also quite present in the *Haplophyllum* genus, in particular scopoletin [56,58,62,68,84,91,98,111]. Coumarins also present chemosystematic relevance in the Rutaceae family [115]. Our results fully confirm this aspect. Flavonoids are widespread secondary metabolites in the plant kingdom with specific functions and in less cases, they have chemotaxonomic relevance. Some of these are rare derivatives with peculiar functionalizations such as that observed for the 8-hydroxyflavone acetylated glycosides that own a restricted distribution among some genera of the Lamioideae subfamily of Lamiaceae, e.g., *Pogostemon*, *Sideritis*, *Stachys*, and *Galeopsis* [116,117,118,119,120,121]. In these genera, isoscutellarein and hypolaetin glycosides have been recognized with glucose and allose as saccharidic moieties. Similarly, it seems that the presence of acetylated 8-hydroxyflavone derivatives related to haplogenin might have a chemotaxonomic relevance given that they represent quite common compounds in the *Haplophyllum* genus. The functionalizations in these 8-hydroxyflavone derivatives involved the presence of glucose and rhamnose as saccharidic units like in haplosides A, B, C, D and limocitrin-7-*O*-β-D-(6″-*O* acetyl)-glucoside [65,68,71,93,94]. In fact, haplosides B and D have been observed in *H. dauricum*, which is one of the few accepted species in the genus, but compounds related to haploside have also been recognized in other *Haplophyllum* species which are of unresolved classifications [65,68,71,93]. Further studies on the phytochemistry of other *Haplophyllum* spp. with a problematic classification are desirable in the future since the distribution of these flavonoids might be of help for their correct classification. The other classes of natural compounds observed in the *Haplophyllum* genus were triterpenoids with β-sitosterol as the major compound [47,49,67,80,99] and lignans with diphyllin as the major compound [49,51,58,65,96,106,111] together with some phenolic acid derivatives. These classes have little chemotaxonomic relevance since they can be biosynthesized by many other plant genera and species such as those belonging to the Araucariaceae [4], Lamiaceae [122], and Orobanchaceae [123] families. Yet, the presence of ferulic acid from *H. foliosum* [69] should be underlined since it is the biogenetic precursor of coumarins. In addition, it is noteworthy that several lignans have been described for the first time in *Haplophyllum*, and these compounds might have a chemotaxonomic relevance. However, further studies are still necessary to confirm this hypothesis.

## 3. Ethnobotany and Biological Activities

The use of many *Haplophyllum* species in traditional medicine has a long history in several countries of the world due to their significant pharmacological activities. In the subsections, the specific ethnobotanical uses and pharmacological properties of *Haplophyllum* species are presented and discussed as well as the pharmacological studies carried out on its components.

### 3.1. H. acutifolium

The paste derived from its whole plant is used in the Iranian northern region of Turkmen Sahra to treat dermal wounds and inflammations [124]. Its ethanolic extract has been reported to be highly and moderately active as cytotoxic agent against RAMOS, MCF-7, and U937 cancer cell lines with IC_50_ values equal to 23.7, 83.5, and 55.9 µg/mL, respectively. This effect is most probably due to the high presence of alkaloids in this plant [125]. In addition, two of its constituents, the alkaloids acutine and haplacutine E, isolated by preparative-scale HPLC, exhibited moderate antiplasmodial activities with IC_50_ values equal to 2.17 μM and 3.79 μM, respectively [43]. Eudesmin isolated from this species also showed good germicidal activity against *Candida albicans*, *Aspergillus flavus*, *Salmonella typhi*, *Klebsiella pneumonia*, and *Fusarium oxysporum*, with growth inhibition percentages well above 50% [46]. Indeed, haplotyn-A, one of its other constituents, showed medium germicidal activity against *Candida albicans*, *Salmonella typhi*, and *Klebsiella pneumoniae*, with growth inhibition percentages between 30 and 40%, except for *K. pneumoniae*, where the value was found to be 51% [46].

### 3.2. H. canalicatum

The methanolic extract of *H. canalicatum* from Iran exhibited moderate cytotoxic activities against several cancer cell lines, e.g., HepG-2, MCF-7, MDBK, WEHI, and A-549, with IC_50_ values higher than 50 μg/mL [126]. This effect has been observed to be mainly due to the quinolinone alkaloids reported in this species. In fact, 7-isopentenyloxy-γ-fagarine, atanine, skimmianine, flindersine, and perfamine were singularly tested for their cytotoxic properties against several cancer cell lines, i.e., HepG-2, MCF, KG-1a, RAJI, and JURKAT, and showed good results. In this context, 7-isopentenyloxy-γ-fagarine was found to be the most active, with IC_50_ values against JURKAT, RAJI, and MCF-7 of 3.6, 1.5, and 15.5 μg/mL, respectively. These values are below the positive control of doxorubicin. In addition, the other compounds have proved to be active even if with a moderate effect. Atanine was found to be more powerful than doxorubicin only against JURKAT (IC_50_ = 9.3 μg/mL). Instead, skimmianine, flindersine, and perfamine were always less potent than doxorubicin against each tested cancer cell line [125]. In addition to this, two other alkaloids isolated from this species, namely acutine and hapacutine E, showed moderate in vitro antiplasmodial activity against chloroquine-sensitive Pfc (3D7 strain), with IC_50_ values of 2.17 and 3.79 µM, respectively [43].

### 3.3. H. myrtifolium

*H. myrtifolium* is used to treat warts, herpes, lichens, erysipelas, diarrhea, and some types of tumors such as testicular cancer [125]. Moreover, its ethanolic extract was found to be a potent antileishmanial agent against the species *Leishmania tropica*, with an IC_50_ value of 10.9 μg/mL [127]. The same effect was also observed for two of its alkaloid constituents, i.e., skimmianine and γ-fagarine, which showed IC_50_ values equal to 25.7 and 8.7 μg/mL, respectively [127]. Moreover, the aerial parts of this species extracted using several solvents proved to possess strong α-glucosidase and α-amylase activities as well as strong anti-acetyl cholinesterase and antidiabetic properties [128].

### 3.4. H. perforatum

*H. perforatum* Kar & Kir. displayed good antimicrobial activities against *Bacillus subtilis*, *Klebsiella pneumoniae*, *Morganella morganti*, and *Staphylococcus aureus* [129]. Moreover, a paste prepared from the aerial parts of *H. perforatum* Kar & Kir. is used by the local people in the Southern regions of Shiraz, Iran, to relieve severe toothaches [130]. It is also noteworthy that the methanolic extract of the leaves of *H. perforatum* Kar & Kir. has potent antifungal activity against *Botrytis cinerea* and *Alternaria solani*. The percentages of growth inhibition were found to be 76.32 and 55.44%, respectively [131]. Indeed, the alkaloids perforine and khaplamine isolated from this species grown in Azerbaijan have been reported to have sedative action [132].

### 3.5. H. sieversii

Two different crude extracts of the aerial parts of *H. sieversii* (petroleum ether and water) were found to have antifungal activity against *Colletotrichum acutatum* Simmonds, *C. fragariae* Brooks, and *C. gloeosporioides* (Penz.) Penz. and Sacc., with inhibition zone diameters below 10 mm [100]. Flindersine and haplamine showed antialgal activity against *Oscillatoria perornata* Skuja with IC_50_ values, after 24 h, equal to 15.9 and 1.8 μM, respectively. These two compounds were found to be also active against *Selenastrum capricornutum* even if with lower IC_50_ values (17.8 and 15.9 μM, respectively). Haplamine was also found to be active against *Pseudanabaena* LW397 having an IC_50_ value of 2.0 μM after 24 h [100].

### 3.6. H. tuberculatum

*H. tuberculatum* has been used in Saudi Arabia for the cure of rheumatoid arthritis, malaria, headaches, and some gynecological problems, as well as to remove warts and freckles from the skin and to treat skin discoloration, infections, and parasitic diseases [133,134]. It is also used in Sudan and Mongolia for the treatment of diarrhea and as an antipyretic agent [135]. In Sudan, the herb is also employed as an antispasmodic, to treat allergic rhinitis, gynecological disorders, asthma, and breathing difficulties [136]. In Algeria, it has been used as an antiseptic, calming, vermifuge, and hypnotic neurological and against injuries, ulcers, infertility, diabetes, bloating, fever, liver diseases, otitis, rheumatism, obesity, constipation, colon, diarrhea, gases, hypertension, menstrual pains, cardiac diseases, scorpion stings, flu, vomiting, throat inflammation, tonsillitis, cough, and loss of appetite [137]. In the northern regions of Oman, the juice made with the leaves has been used to treat headaches and arthritis for many years [138]. In Egypt, the flowering parts are used as a drink to treat fever, abdominal upset, anemia, gastric pains, intestinal worms, malaria, and as an aphrodisiac, while its decoction is used for rheumatic pains [139]. Moreover, its ethanolic extract was observed to have high cytotoxic activities against RAMOS, U937, MCF-7, LNCap-FGC-10, 5637, and RPMI-8866 cancer cell lines. The relative IC_50_ values were 25.3, 29.3, 57.2, below 7.81, 23.3, and 31.8 µg/mL, respectively. This effect is mainly due to its alkaloid content [125]. The same extract is also able to exhibit strong antimicrobial, anti-inflammatory and antifungal effects [136]. A strong effect was also observed for the essential oil derived from the aerial parts against *Aedes aegypti*. In particular, as reported, this oil could kill 100% of its larvae at 250 and 125 ppm [34]. In addition, a medium germicidal effect was observed for the same essential oil against several microorganisms such as *Candida* spp., *Alternaria alternata*, *Curvularia lunata*, *Fusarium oxysporum*, *Stemphylium solani*, and *Aspergillus flavus* with MIC values below 1 mg/mL [32]. Indeed, against *Escherichia coli*, *Staphylococcus aureus*, *Salmonella choleraesuis*, and *Bacillus subtilis*, the inhibition zone diameters were 17.6, 6.7, 17.3, and 12.3 mm, respectively. The *n*-hexane extract of this species also showed medium antibacterial effects against *Staphylococcus aureus*, *Escherichia coli* and *Pseudomonas aeruginosa*, with inhibition zone diameters of 12, 10, and 16 mm, respectively. The chloroform and methanol extracts were active, in this sense, only against *Pseudomonas aeruginosa*, with inhibition zone diameters of 11 and 17 mm, respectively [35]. The main responsible compounds for this seem to be the alkaloids and the lignans. The essential oil is also able to exhibit good antitumor activities against lung carcinoma H1299 cell lines, with an IC_50_ value equal to 4.7 μg/mL [37]. The aqueous extract of the leaves has also antispasmodic effects [140]. Additionally, one of its constituents, the alkaloid tuberine, has shown high anti-microbial activity against *Bacillus subtilis* and *Saccharomyces cerevisiae* at the concentration of 1 μg/mL [141]. Another alkaloid constituent, dihydroperfamine, was found to have strong anxiolytic effects [103]. Indeed, one of its lignans, 1-hydroxy-3-(hydroxymethyl)-6,7-dimethoxy-4-(3,4-methylenedioxyphenyl)-2-naphthoic acid γ-lactone, has shown good selective antitumor effects against the human lung cancer cell lines H-125M, with inhibition zone units equal to 700 [109]. Lastly, its lignans justicidin A, justicidin B, tuberculatin, and acetyl-tuberculatin possess strong cytotoxic effects against A375 cancer cell lines with GI_50_ values equal to 25, 17, 3, and 3 μM, respectively [110]. Unfortunately, it is quite important to highlight that the species is severely threatened and is at the verge of extinction in some countries [142].

### 3.7. Other Species

The lignan diphyllin, isolated from *H. bucharicum*, exhibited strong antileishmanial activity, especially against intracellular amastigote forms (IC_50_ = 0.2 μM), while it did not show remarkable activity against the promastigote forms (IC_50_ = 14.4 μM). Moreover, it possesses moderate antiproliferative effects on human monocytes, with an IC_50_ value of 35.2 μM [143].

*H. dauricum* is employed mainly in Mongolia as an antitumor agent [144], especially because of its coumarin content [145]. In addition, one of its lignan components, daurinol, has shown remarkable cytotoxic properties (IC_50_ below 20 µM), being a potential catalytic inhibitor of topoisomerase IIα and acting at the S phase, thus not causing DNA or RNA damages [146,147].

*H. leptomerum* is used in Uzbekistan for its cytotoxic activities [148], mainly due to one of its constituents, the alkaloid dictamine, which is able to exhibit strong cytotoxic effects against the human cancer cell lines, e.g., HeLa and HCT-116, with IC_50_ values equal to 65.0 and 85.0 μM, respectively [81].

*H. pedicellatum* has shown to possess antimicrobial activity against *Pseudomonas aeruginosa* [129].

The lignan 1β-polygamain from *H. ptilosyylum* showed strong cytotoxic activity (IC_50_ = 111.7 pg/mL) against HIV-1 [95].

The infusion of *H. robustum* whole plant is frequently used in the Iranian northern region of Maraveh Tappeh against dermal wounds as a beverage, thus acting from the inside [149].

The ethanolic extract of *H. stapfanum* Hand.-Mazz. displayed high cytotoxic properties against RAMOS, U937, and LNCap-FGC-10 cancer cell lines (IC_50_ values are equal to 12.3, 15.6, and 28.3 µg/mL, respectively), as well as a moderate activity against the 5637 and MCF-7 cancer cell lines (IC_50_ values are equal to 23.3 and 92.6 µg/mL, respectively). These effects are thought to be due to its alkaloid content, but no precise phytochemical analysis has been conducted on this species up to present [125].

*H. telephioides* is used in some areas of Turkey to treat flu [150].

*H. tenue* ethanolic extract and EO showed high radical scavenging activity, with IC_50_ values equal to 103.88 and 101.98 pg/mL, respectively. In addition, the ethanolic extract showed strong antimicrobial activity against *Clostridium perfringens* (IC_50_ = 16 pg/mL) [151].

Lastly, the ethanolic extract of *H. viridulum* Soják from Iran displayed moderate cytotoxic activities against RAMOS and U937 cancer cell lines, with IC_50_ values of 48.3 and 79.0 µg/mL, respectively) [125].

## 4. Conclusions

In the current review paper, the literature data have been systematically reviewed and different aspects relating to the numerous *Haplophyllum* species have been discussed.

From a phytochemical point of view, a large number of bioactive natural compounds, both volatile and non-volatile, have been characterized. In addition, as discussed earlier, the ethnobotanical knowledge of *Haplophyllum* species is valuable, and these species are widely prescribed in the traditional medicine of many countries, in particular in the Middle East. The other aspect of *Haplophyllum* which deserves more attention is the growing interest to study the potential biological activities of its species. In this sense, *Haplophyllum* species, as well as their bioactive compounds, are able to exhibit many pharmacological activities, among which the cytotoxic, antiviral, antifungal and antimicrobial are the most important. However, it should be underlined that further investigations are still required to confirm the real therapeutic potentials of these species and to represent their remarkable phytochemical and biological potency. Summarizing, the tabulated and argued data in the current review paper can attract the attention of the scientific community towards the *Haplophyllum* species and prompt researchers in phytochemical, pharmaceutical, and related areas to design and develop more attempts on these valuable herbal plants.

## Figures and Tables

**Figure 2:**
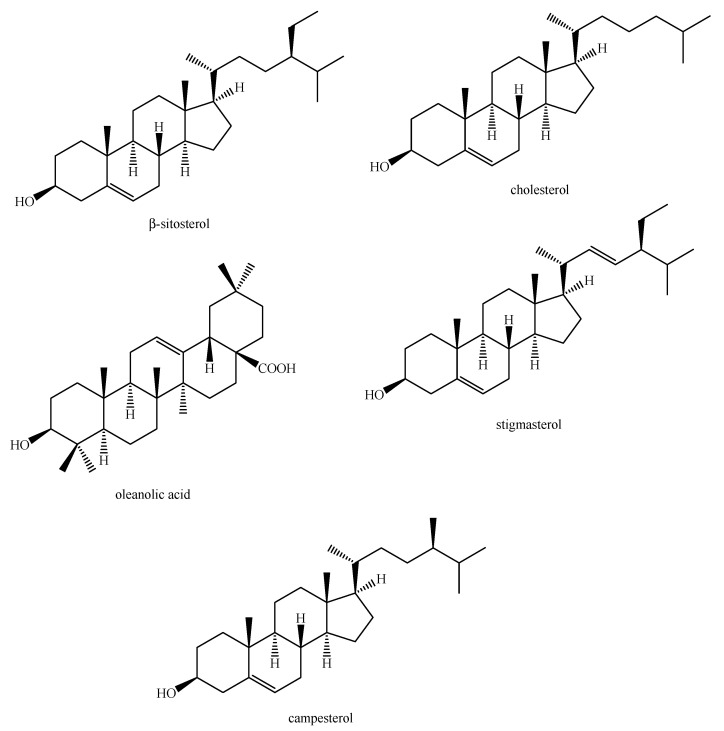
Structure of the terpenoids identified in *Haplophyllum* species.

**Figure 3:**
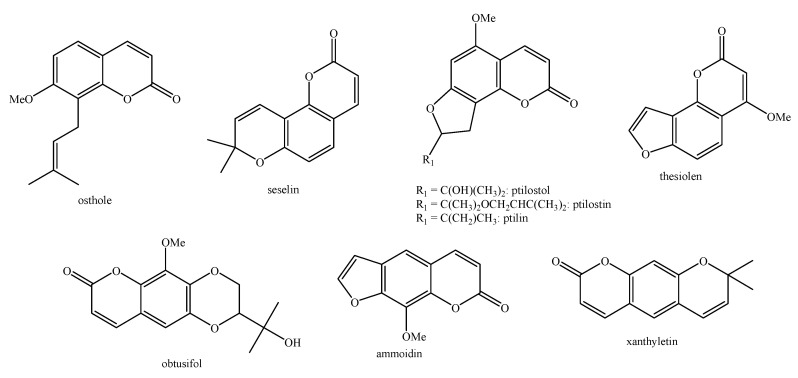
Structure of the coumarins identified in *Haplophyllum* species—part 1.

**Figure 4:**
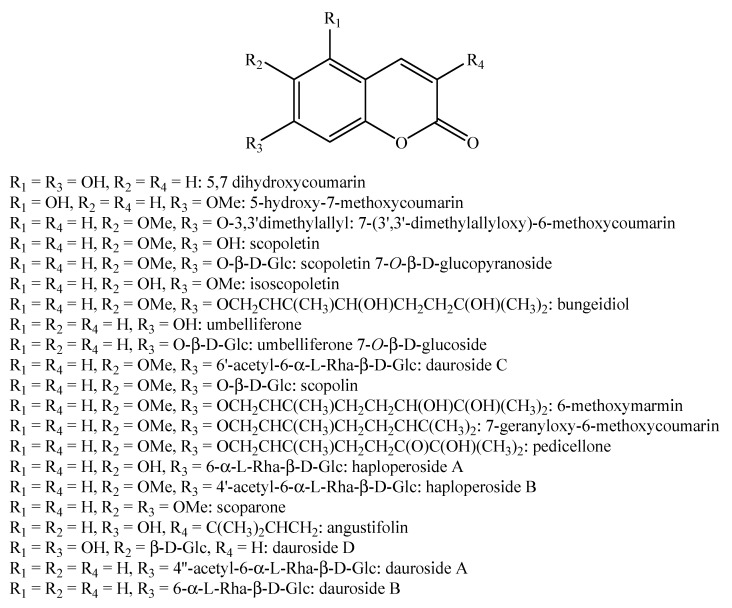
Structure of the coumarins identified in *Haplophyllum* species—part 2.

**Figure 5:**
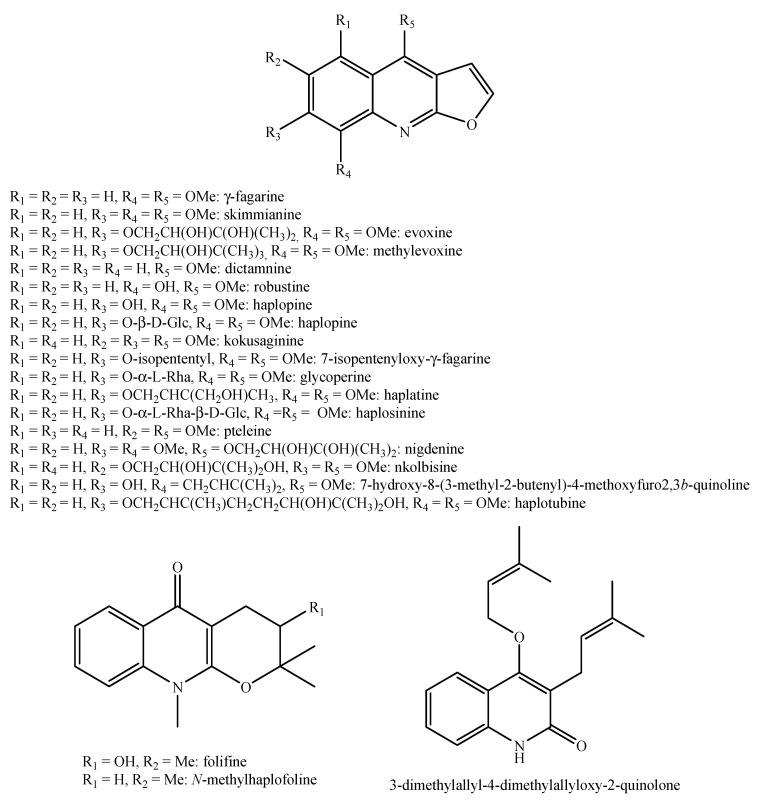
Structure of the alkaloids identified in *Haplophyllum* species—part 1.

**Figure 6:**
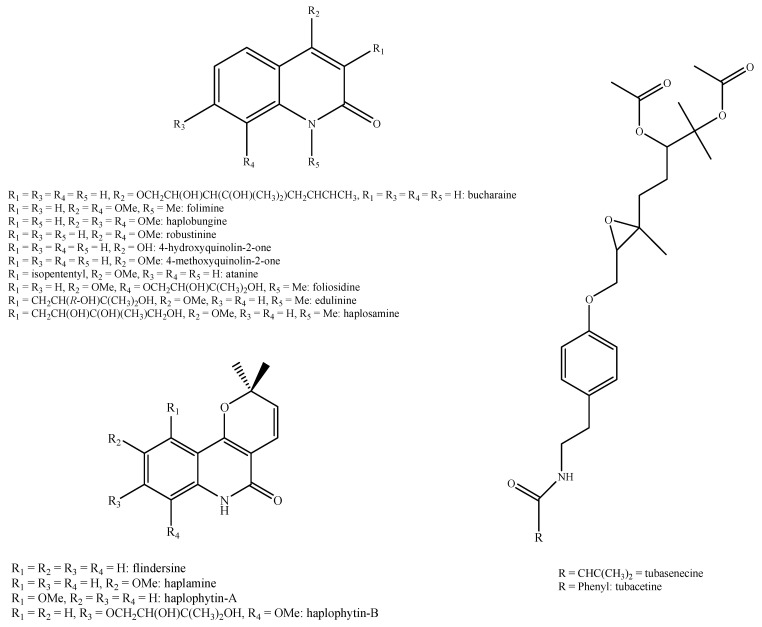
Structure of the alkaloids identified in *Haplophyllum* species—part 2.

**Figure 7:**
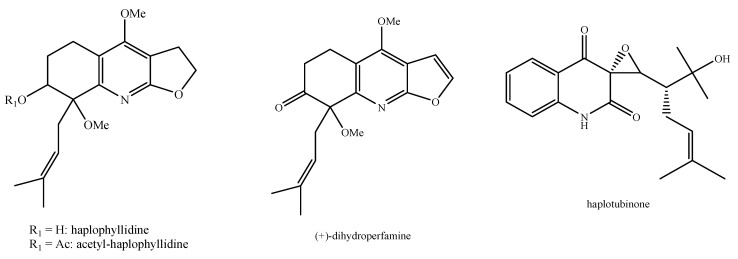
Structure of the alkaloids identified in *Haplophyllum* species—part 3.

**Figure 8:**
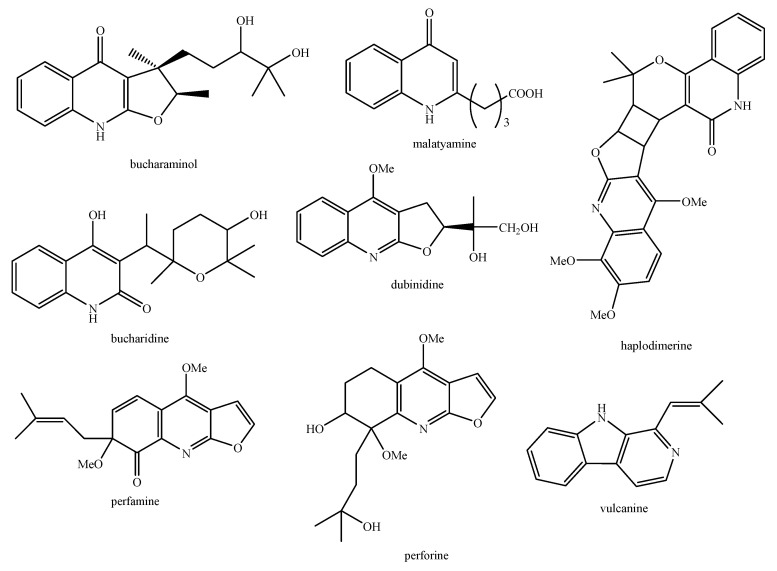
Structure of the alkaloids identified in *Haplophyllum* species—part 4.

**Figure 9:**
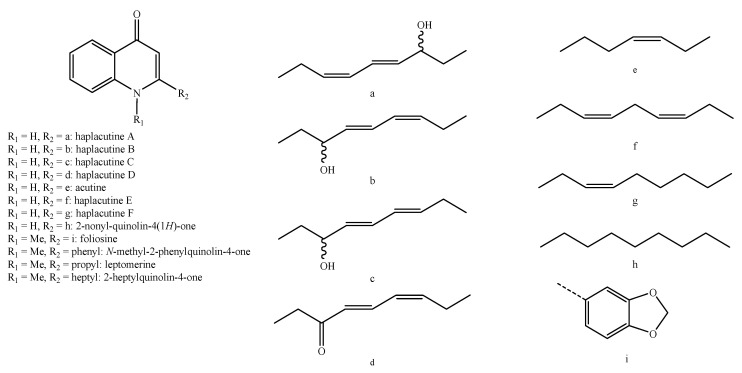
Structure of the alkaloids identified in *Haplophyllum* species—part 5.

**Figure 10:**
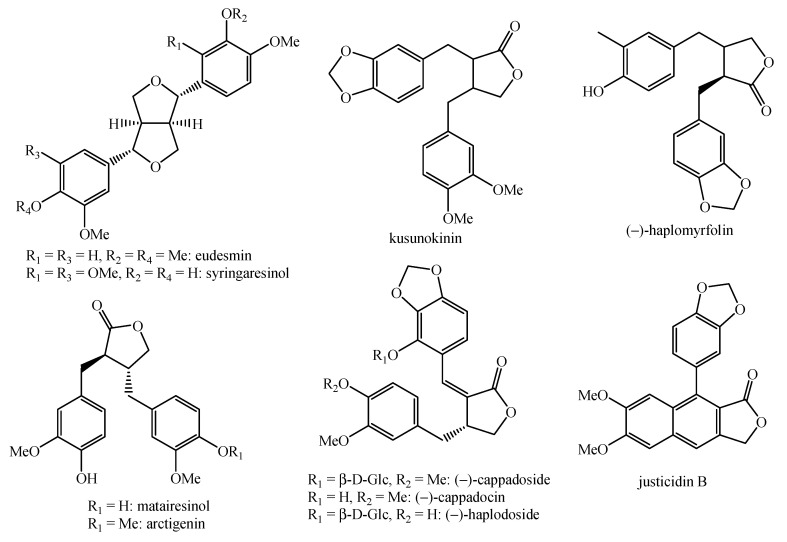
Structure of the lignans identified in *Haplophyllum* species—part 1.

**Figure 11:**
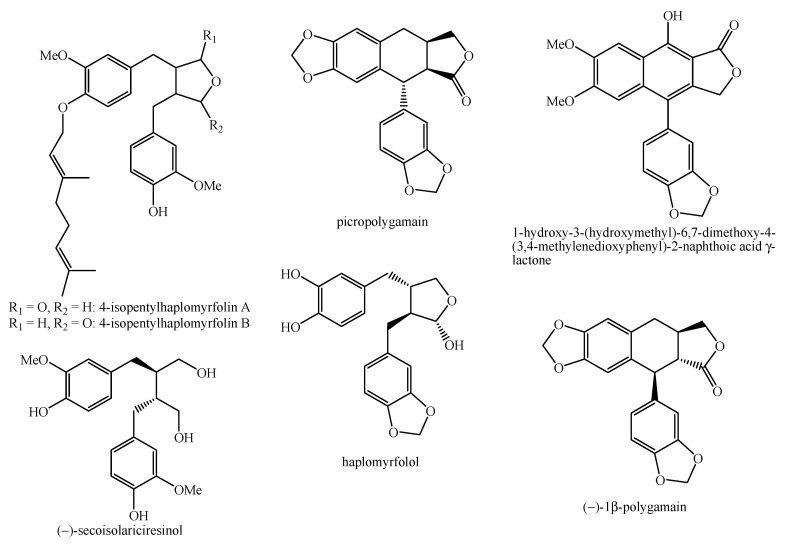
Structure of the lignans identified in *Haplophyllum* species—part 2.

**Figure 12:**
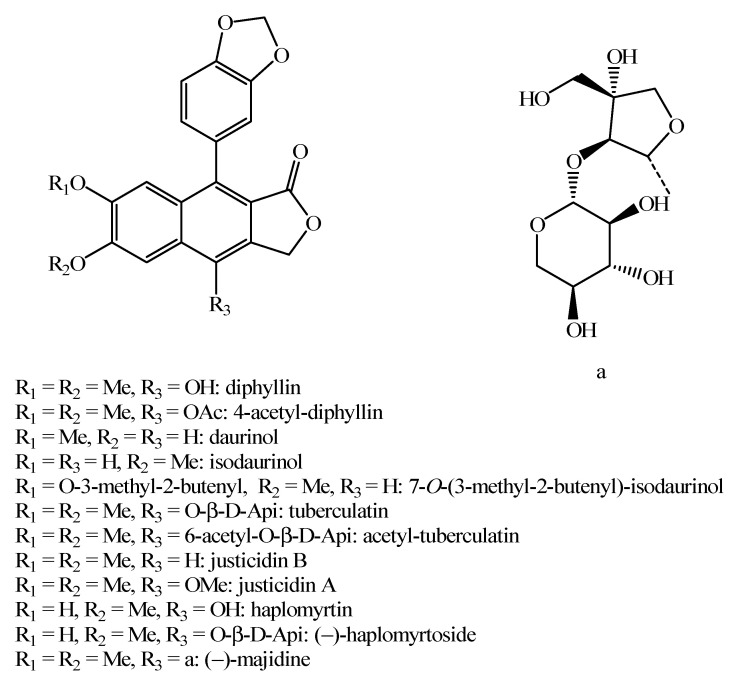
Structure of the lignans identified in *Haplophyllum* species—part 3.

**Figure 13:**
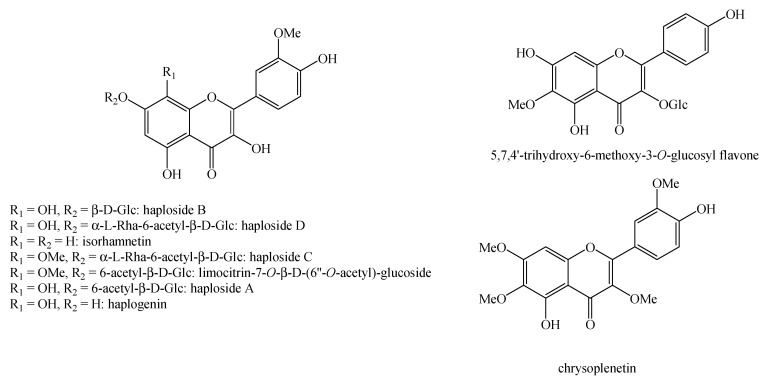
Structure of the flavonoids identified in *Haplophyllum* species.

**Figure 14:**
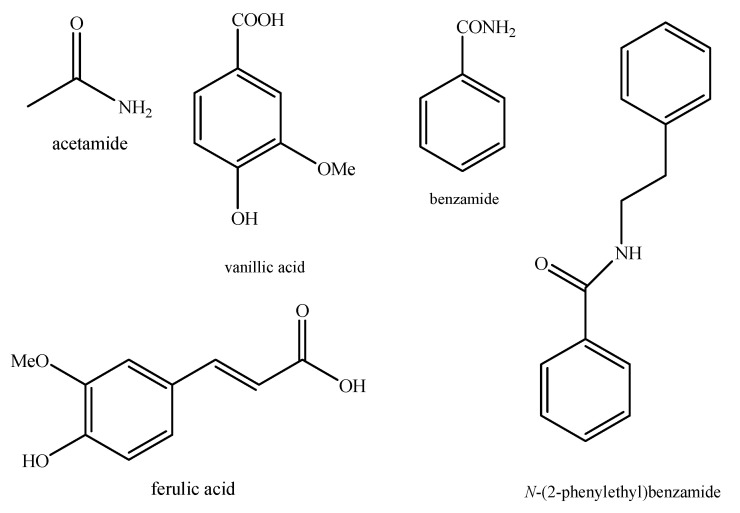
Structure of the other compounds identified in *Haplophyllum* species.

**Table 1:** Main volatile constituents from different species of *Haplophyllum* genus worldwide.

Plant Species	Main Components (%)	OY ^a^	Identified Compounds		Dominant Group	Extraction Method	Analysis Method	Studied Organs	Country	Reference
			Nr.	%						
*H. acutifolium (DC.) G. Don*	α-Cadinene (25.1%), β-cedrene (19.1%), sabinene (8.1%), terpinen-4-ol (5.7%), and 8,14-cedranoxide (5.5%)	0.1	92	97.7	SH ^b^	CHD ^c^	GC, GC-MS	Aerial parts	Iran	[16]
*H. buhsei* Boiss.	β-Caryophyllene (12.9%), limonene (9.7%), β-pinene (7.9%), linalool (7.4%), α-pinene (6.4%), and 1,8-cineole (5.5%)	0.35	36	92.2	MH ^d^	CHD	GC, GC-MS	FAP ^e^	Iran	[17]
*H. furfuraceum* Bunge	Elemol (11.7%), β-eudesmol (10.1%), 1,8-cineole (9.3%), α-pinene (8.5%), β-pinene (7.7%), caryophyllene oxide (5.9%), and *p*-cymene (5.2%)	0.35	33	98.1	MH~OS ^g^	CHD	GC, GC-MS	Aerial parts	Iran	[18]
*H. glaberrimum* Bunge	Myrcene (52.9%), elemol (10.6), and β-caryophyllene (8.9%)	0.08	10	93.9	MH	CHD	GC, GC-MS	Leaves	Iran	[19]
	Myrcene (65.1%), α-thujene (5.4%), and *trans*-β-ocimene (4.7%)	0.14	16	96.9				Aerial parts		
*H. laeviusculum* C. C. Towns.	β-Pinene (20.1%), α-phellandrene (11.7%), β-caryophyllene (7.6%), myrcene (6.8%), linalool (6.1%), and limonene (5.6%)	NA ^h^	36	95.7	MH	CHD	GC, GC-MS	FAP	Iran	[20]
*H. lissonotum* C.C. Towns.	Caryophyllene oxide (26.9%), β-caryophyllene (12.2%), humulene epoxide II (8.3%), α-caryophyllene (7.2%), and caryophylla-4(14),8(15)-dien-5β-ol (7.1%)	0.23	50	88.5	OS	CHD	GC, GC-MS	Aerial parts	Iran	[21]
*H. megalanthum* Bornm.	Palmito-γ-lactone (45.8%), octadecatrienoic acid (10.7%), linoleic acid (6.5%), octadecatetraenoic acid (6.3%), and nonacosane (4.8%)	0.1	58	91.7	NH	CHD	GC, GC-MS	FAP	Turkey	[22]
*H. myrtifolium* Boiss.	PEE ^l^: β-Caryophyllene (14.6%), decane (11.4%), and β-phellandrene (7.0%)	-	47	69	-	SPME ^n^	GC-MS	Aerial parts	Turkey	[23]
	CEAE ^m^: Havibetol (21.9%), eugenol (19.1%), methyl- eugenol (10.8%), *trans*-linalool oxide (7.1%), and β-cyclocitral (6.0%)		42	83.2	NH					
	Linalool (12.8%), β-caryophyllene (10.3%), and methyl eugenol (5.9%)	NR	97	85.3	-	CHD	GC-MS	Aerial parts	Turkey	[24]
*H. perforatum* Kar. & Kir.	Sabinene (52.7%), β-caryophyllene (10.8%), (2*E*,6*E*)-farnesyl acetone (10.3%), hexadecanoic acid (5.1%), β-pinene (5.0%), and *cis*-sabinene hydrate (4.9%)	-	9	95.9	MH	HS-SPME ^o^	GC, GC-MS	Flowers	Iran	[25]
	Sabinene (24.7%), β-caryophyllene (35.6%), elemol (17.4%), α-caryophyllene (4.6%), α-pinene (4.5%), and 1,8-cineole (4.3%)		10	99.7	SH			Leaves		
	Sabinene (26.2%), β-caryophyllene (8.8%), camphor (7.4%), limonene (6.3%), elemol (5.0%), β-phellandrene (4.9%), and α-pinene (4.6%)		19	81.3	MH			Stems		
*H. robustum* Bunge	Sabinene (30.5%), β-pinene (18.2%), and limonene (12.1%)	0.5	23	86.1	MH	CHD	GC-MS	Aerial parts	Iran	[26]
	1,8-Cineole (38.1%), myrcene (10.7%), α-pinene (8.5%), terpinen-4-ol (7.0%), and sabinene (6.1%)		30	99.2	OM ^p^			Whole plant		[27]
	*cis*-Sabinene hydrate (23.2%), 1,8-cineole (19.1%), γ-terpinene (10.3%), limonene (7.3%), and β-pinene (6.1%)	1.1	13	82.7			GC, GC-MS	Leaves		[28]
	1,8-Cineole (27.7%), γ-terpinene (12.2%), *cis*-sabinene hydrate (11.5%), limonene (11.1%), and β-pinene (7.7%)	0.39	12	82.7				Stems		
	1,8-Cineole (45.1%), limonene (12.3%), *cis*-sabinene hydrate (12.0%), γ-terpinene (6.7%), and β-pinene (6.1%)	1.1	11	89.2				Flowers		
	1,8-Cineole (28.4%), limonene (13.8%), *cis*-sabinene hydrate (12.2%), γ-terpinene (10.1%), and β-pinene (8.7%)	2.1	12	83.4				Fruits		
	1,8-Cineole (38.1%), myrcene (10.7%), α-pinene (8.5%), terpinen-4-ol (7.0%), sabinene (6.2%), methyl-geranate (4.7%), γ-terpinene (4.3%), and α-terpinene (3.4%)	0.5	30	99.2	OM	CHD	GC, GC-MS	Aerial parts	Iran	[29]
*H. tuberculatum* Juss	Limonene (27.3%), and α-pinene (21.9%)	0.35	18	79.7	MH	CHD	GC, GC-MS	Aerial parts	Iran	[30]
	α-Phellandrene (10.7-32.9%), β-caryophyllene (6.3-12.8%), β-pinene (7.6-8.0%), limonene (4.0-9.6%), and δ-3-carene (5.5-6.0%) ^q^	0.03	23 ^r^	80.2 ^r^	MH	CHD		FAPCF ^u^	United Arab Emirates	[31]
			29 ^s^	78.7 ^s^						
	Linalool (15.0%), linalyl acetate (10.6%), β-caryophyllene (9.7%), and α-terpineol (6.7%) ^t^	0.04	28	77.4	OM					
	β-Phellandrene (23.3%), limonene (12.6%), β-ocimene (12.3%), β-caryophyllene (11.6%), myrcene (11.3%), and α-phellandrene (10.9%)	0.21	30	99.7	MH		GC-MS, ^13^C NMR	FTF ^v^	Oman	[32]
	Linalool (15.5%), α-pinene (7.9%), and limonene (5.3%)	0.02	40	98.1			GC, GC-MS	Aerial parts	Iran	[33]
	*trans*-*p*-Menth-2-en-1-ol (19.2%), *cis*-*p*-menth-2-en-1-ol (13.2%), myrcene (10.1%), δ-3- carene (8.8%), β-phellandrene (6.9%), limonene (6.6%), *cis*-piperitol (6.4%), piperitone (4.1%), and *trans*-piperitol (4.0%)	NR	37	96.4	OM					
								FAP	Saudi Arabia	[34]
	Hexadecanoic acid (40.2%) and oleic acid (26.8%)	1.54	18	93.5	NH			Shoots	Tunisia	[35]
	2,4-*Bis*(1,1-dimethylethyl)-phenol (28.3%), piperitone (17. 8%), terpinen-4-ol (3.2.%), hexadec-1-ene (3.2%), β-phellandrene (3.0%), *p*-cymene-8-ol (2.9%), (1*E*,4*E*)-germacrene B (2.1%), octadec-1-ene (2.1%), and α-phellandrene (2.1%)	0.91	26	82.5	OM			Aerial parts	Algeria	[36]
	α-Terpinene (26.4%), β-terpinene (17.1%), β-phellandrene (10.4%), γ-terpinene (9.1%), 3,7-dimethyl-cyclooctadiene (6.0%), and myrcene (5.7%)	0.4	24	95.8	MH		GC-FID, GC-MS	Aerial parts	Egypt	[37]
	α-Terpinene (24.4%), β-terpinene (14.4%), β-phellandrene (10.0%), γ-terpinene (7.8%), 3,7-dimethyl-cyclooctadiene (6.7%), and myrcene (6.0%)	1.5	28	97.0				Flowers		
	*cis*-*p*-Menth-2-en-1-ol (16.8%), *trans*-*p*-menth-2-en-1-ol (16.2%), *trans*-piperitol (12.1%), limonene (8.1%), piperitone (6.7%) 1-octyl acetate (5.4%), and *cis*-piperitol (4.9%)	NR	32	94.4	OM		GG-MS	Leaves	Tunisia	[38]
	Isobornyl acetate (13.8%), *cis*-*p*-menth-2-en-1-ol (12.4%), *trans*-*p*-menth-2-en-1-ol (11.2%), *trans*-piperitol (9.1%), piperitone (8.5%), 1-octyl acetate (7.4%), α-pinene (4.6%), and *cis*-piperitol (4.0%)		24	94.3				Stems		
	Piperitone (9.1%), 1-octyl acetate (8.8%), *cis*-*p*-menth-2-en-1-ol (8.7%), *trans*-*p*-menth-2-en-1-ol (8.2%), isobornyl acetate (7.8%), *trans*-piperitol (5.5%), limonene (5.2%), cryptone (4.5%), and α-pipene (3.9%)		37	91.3				Leaves and stems		
*H. virgatum* Spach.	2-Nonanone (28.4%), 2-undecanone (21.5%), 1,8-cineole (9.5%), caryophyllene oxide (6.8%), and linalool (5.0%)	0.2	25	90.5	NH	CHD	GC, GC-MS	Aerial parts	Iran	[18]
	Valencene (14.6%), β-pinene (13.1%), limonene (8.8%), δ-3-carene (8.2%), aromadendrene (8.1%), and piperitone (6.8%)	0.3	39	95.9	MH		GC-MS			[39]

^a^ OY: Oil yield; ^b^ SH: Sesquiterpene hydrocarbon; ^c^ CHD: Classical hydrodistillation; ^d^ MH: Monoterpene hydrocarbon; ^e^ FAP: Flowering aerial parts; ^g^ OS: Oxygenated sesquiterpene; ^h^ NA: Not available; ^l^ PEE: Petroleum ether extract; ^m^ CEAE: Chloroform eluate of the alkaloidal extract; ^n^ SPME: Solid phase microextraction; ^o^ HS-SPME: Head space-solid phase microextraction; ^p^ OM: Oxygenated monoterpene; ^q^ Plants collected in May (1997 and 2001); ^r^ May (1997); ^s^ May (2001); ^t^ Plants collected in April (1998); ^u^ FAPIF: Fresh aerial parts, including flowers; ^v^ FTF: Fresh twigs and flowers.

**Table 2:** Distribution of the main volatile phytochemicals in the *Haplophyllum* genus.

Phytochemical Class	Phytochemical Compound	*Haplophyllum* spp.	References
Monoterpene hydrocarbons	α-Phellandrene	*H. laeviusculum* *H. tuberculatum*	[20,31,32,36]
	α-Pinene	*H. buhsei* *H. furfuraceum* *H. perforatum* *H. robustum* *H. tuberculatum*	[17,18,25,27,28,29,30,33,38]
	α-Terpinene	*H. robustum* *H. tuberculatum*	[29,37]
	α-Thujene	*H. glaberrimum*	[19]
	β-Ocimene	*H. tuberculatum*	[32]
	β-Phellandrene	*H. myrtifolium* *H. perforatum* *H. tuberculatum*	[23,25,32,34,36,37]
	β-Pinene	*H. buhsei* *H. furfuraceum* *H. laeviusculum* *H. perforatum* *H. robustum* *H. tuberculatum* *H. virgatum*	[17,18,20,25,26,28,31,39]
	β-Terpinene	*H. tuberculatum*	[37]
	γ-Terpinene	*H. robustum* *H. tuberculatum*	[28,29,37]
	δ-3-Carene	*H. tuberculatum* *H. virgatum*	[31,34,39]
	*p*-Cymene	*H. furfuraceum*	[18]
	*Cis*-sabinene hydrate	*H. perforatum* *H. robustum*	[25,28]
	Isobornyl acetate	*H. tuberculatum*	[38]
	Limonene	*H. buhsei* *H. laeviusculum* *H. perforatum* *H. robustum* *H. tuberculatum* *H. virgatum*	[17,20,25,26,28,29,30,31,32,33,34,38,39]
	Myrcene	*H. glaberrimum* *H. laeviusculum* *H. robustum* *H. tuberculatum*	[19,20,27,32,34,37]
	Sabinene	*H. acutifolium* *H. perforatum* *H. robustum*	[16,25,26,27,29]
	*Trans*-β-ocimene	*H. glaberrimum*	[19]
Non-terpene hydrocarbons	1-Octyl acetate	*H. tuberculatum*	[38]
	2,4-*Bis*(1,1-dimethylethyl)-phenol	*H. tuberculatum*	[36]
	3,7-Dimethyl-cyclooctadiene	*H. tuberculatum*	[37]
	(2*E*,6*E*)-Farnesyl acetone	*H. perforatum*	[25]
	2-Nonanone	*H. virgatum*	[18]
	2-Undecanone	*H. virgatum*	[18]
	β-Cyclocitral	*H. myrtifolium*	[23]
	Decane	*H. myrtifolium*	[23]
	Eugenol	*H. myrtifolium*	[23]
	Havibetol	*H. myrtifolium*	[23]
	Hexadec-1-ene	*H. tuberculatum*	[36]
	Hexadecanoic acid	*H. perforatum* *H. tuberculatum*	[25,35]
	Linoleic acid	*H. megalanthum*	[22]
	Methyl-eugenol	*H. myrtifolium*	[23,24]
	Methyl-geranate	*H. robustum*	[29]
	Nonacosane	*H. megalanthum*	[22]
	Octadec-1-ene	*H. tuberculatum*	[36]
	Octadecatrienoic acid	*H. megalanthum*	[22]
	Octadecatetraenoic acid	*H. megalanthum*	[22]
	Oleic acid	*H. tuberculatum*	[35]
	Palmito-γ-lactone	*H. megalanthum*	[22]
Oxygenated monoterpenes	1,8-Cineole	*H. buhsei* *H. furfuraceum* *H. perforatum* *H. robustum* *H. virgatum*	[17,18,25,27,28,29,39]
	α-Terpineol	*H. tuberculatum*	[31]
	*p*-Cymene-8-ol	*H. tuberculatum*	[36]
	Camphor	*H. perforatum*	[25]
	*Cis*-*p*-menth-2-en-1-ol	*H. tuberculatum*	[34,38]
	*Cis*-piperitol	*H. tuberculatum*	[34,38]
	Cryptone	*H. tuberculatum*	[38]
	Linalool	*H. buhsei* *H. laeviusculum* *H. myrtifolium* *H. tuberculatum* *H. virgatum*	[17,18,20,24,31,33]
	Linalyl acetate	*H. tuberculatum*	[31]
	Piperitone	*H. tuberculatum* *H. virgatum*	[34,36,38,39]
	Terpinen-4-ol	*H. acutifolium* *H. robustum* *H. tuberculatum*	[16,27,29,36]
	*Trans*-*p*-menth-2-en-1-ol	*H. tuberculatum*	[34,38]
	*Trans*-linalool oxide	*H. myrtifolium*	[23]
	*Trans*-piperitol	*H. tuberculatum*	[34,38]
Oxygenated sesquiterpenes	8,14-Cedranoxide	*H. acutifolium*	[16]
	β-Eudesmol	*H. furfuraceum*	[18]
	Caryophyllene oxide	*H. furfuraceum* *H. lissonotum* *H. virgatum*	[18]
	Caryophylla-4(14),8(15)-dien-5β-ol	*H. lissonotum*	[21]
	Elemol	*H. furfuraceum* *H. glaberrimum* *H. perforatum*	[18,19,25]
	Humulene epoxide II	*H. lissonotum*	[21]
Sesquiterpene hydrocarbons	(1*E*,4*E*)-Germacrene B	*H. tuberculatum*	[36]
	α-Cadinene	*H. acutifolium*	[16]
	α-Caryophyllene	*H. lissonotum* *H. perforatum*	[21,25]
	β-Cedrene	*H. acutifolium*	[16]
	β-Caryophyllene	*H. buhsei* *H. glaberrimum* *H. laeviusculum* *H. lissonotum* *H. myrtifolium* *H. perforatum* *H. tuberculatum*	[17,19,20,21,23,24,25,31,32]
	Aromadendrene	*H. virgatum*	[39]
	Valencene	*H. virgatum*	[39]

**Table 3:** Non-volatile compounds evidenced in *Haplophyllum* spp.

Plant Species	Compounds	Extraction Solvent	Analysis Method	Studied Organs	Country	Reference
*H. acutifolium (DC.) G. Don*	Haplacutine A, haplacutine B, haplacutine C, haplacutine D, acutine, haplamine, haplacutine E, haplacutine F, and 2-nonyl-quinolin-4(1*H*)-one	Ethyl acetate	HPLC-PDA-MS, SPE-NMR, UV and IR	Aerial parts	Iran	[43]
	Acutine, skimmianine, and acetamide	Chloroform	CC, UV, TLC, NMR and MS	Epigeal parts	Turkmenistan	[44]
	Skimmianine and evoxine	N.D.	N.D.	N.D.	Tajikistan	[45]
	β-Sitosterol, cholesterol, oleanolic acid, haplophytin-A, haplophytin-B, haplotin, flindersine, and kusunokinin	Methanol	CC, UV, NMR and MS	Whole plant	Pakistan	[46,47]
	Eudesmin	Ethereal eluates	CC, IR, UV, NMR, and MS	Epigeal parts	Uzbekistan	[46,48]
*H. alberti-regelii* Korovin	Diphyllin	Methanol	CC, IR, UV, NMR, and MS		Tajikistan	[49]
*H. boissierianum* Beck	ECNP	Methanol and ethanol	Phytochemical screening	Aerial parts	Serbia	[50]
*H. bucharicum* Litv.	Diphyllin	Methanol	CC, IR, UV, NMR, and MS	Epigeal parts	Tajikistan	[49]
	β-Sitosterol, stigmasterol, campesterol, cholesterol, skimmianine, bucharaine, and 3-dimethylallyl-4-dimethylallyloxy-2-quinoline		CC, IR, NMR, and MS	Aerial parts	Russia (Dagestan republic)	[49]
	Diphyllin, 4-acetyl-diphyllin, bucharaine, skimmianine, bucharaminol, bucharidine, 4-hydroxyquinolin-2-one, 4-methoxyquinolin-2-onem and justicidin B		MP, CC, and NMR		Uzbekistan (different districts)	[51]
	Skimmianine, dictamnine, γ-fagarine, robustine, haplopine, flindersine, and haplamine		MP, CC, and NMR	Roots	Uzbekistan (Surkhandarinskii district)	[51]
	Bucharaine, skimmianine, haplopine, folifine, bucharidine, γ-fagarine, robustine, and benzamide	Chloroform and phenolic partitions	CC, IR, UV, and NMR	Mother liquor from the roots	Turkmenistan	[52]
*H. bungei* Trautv.	Skimmianine, haplopine, haplamine, γ-fagarine and POCS	Methanol	HPLC-UV	Leaves	Uzbekistan	[53]
	Dictamnine, skimmianine, folimine, robustinine, 4-methoxyquinolin-2-one, and haplobungine		CC, UV, IR, MS, NMR, and MP	Epigeal parts	Kazakhstan	[54]
	Osthole, 7-(3′,3′-dimethylallyloxy)-6-methoxycoumarin, and 5-hydroxy-7-methoxycoumarin	Chloroform	MP, IR, and NMR		Turkmenistan	[55]
	Scopoletin, isoscopoletin, and bungeidiol	N.D.	CC, MP, IR, and NMR		Azerbaijan	[56]
*H. canaliculatum* Boiss.	7-Isopentenyloxy-γ-fagarine, atanine, skimmianine, flindersine, and perfamine	Methanol	CC, HPLC-UV, and NMR	Aerial parts	Iran	[57]
*H. cappadocicum* Spach	Isodaurinol, daurinol, justicidin A, justicidin B, diphyllin, matairesinol, dictamnine, robustine, haplopine, skimmianine, scopoletin, and seselin	Ethanol	CC, NMR, UV, and MS	Whole plant	Turkey	[58]
	(−)-Cappadoside, (−)-cappodicin, and (−)-haplodoside		IR, NMR, MS, and UV		Turkey	[59]
	(−)-haplomyrtoside, (−)-majidine, (−)-lβ-polygamain, and vanillic acid		CC, UV, IR, NMR, and MS		Iran	[60]
	Malatyamine		CC, IR, NMR, and MS		Turkey	[61]
*H. dauricum* (L.) G. Don	Justicidin B, daurinol, umbelliferone, umbelliferone 7-*O*-β-D-glucoside, 5,7-dihydroxy-coumarin, and dauroside D		Ripartition in chloroform, CC, IR, UV, NMR, and MS	Epigeal parts	Mongolia	[62]
	Dauroside A and dauroside B		CC, UV, IR, α_D_, NMR, and MS			[63,64]
	Diphyllin, scopoletin, dauroside C, haploside B, and haploside D	N.D.	CC, IR, NMR, and MS	Whole plant	N.D.	[65]
	Robustine, dictamnine, γ-fagarine, haplopine, skimmianine, 4-methoxy-*N*-methyl-2-quinolone, folimine, robustinine, and daurine	Methanol	CC, UV, IR, NMR, and MS	Roots	Mongolia	[66]
*H. dshungaricum* Rubtzov	Seselin and xanthyletin	Ethanol	CC, TLC, MP, IR, and NMR	Whole plant	Kazakhstan	[67]
*H. dubium* Korovin	Scopoletin, scopolin, haploside B, and haploside D		CC, MP, UV, NMR, and MS	Epigeal parts	Tajikistan	[68]
*H. foliosum* Vved.	Foliosidine, haplodimerine, skimmianine, *N*-methyl-2-phenyl-4-quinolone, foliosine, and folimine	Chloroform	CC, IR, UV, NMR, and MS		N.D.	[46]
	Folimine, foliosidine, dubinidine, foliosine, edulinine, folidine, and ferulic acid	Methanol	CC, IR, UV, NMR, and MS		Tajikistan	[69,70]
	Isorhamnetin, haploside C, and limocitrin-7-*O*-β-D-(6″-*O* acetyl)-glucoside	Ethanol	CC, UV, NMR, and MS	Aerial parts	Kyrgyzstan	[71]
*H. glaberrimum* Bunge	ECNP	N.D.	Phytochemical screening		Uzbekistan	[72]
*H. griffithianum* Boiss.	Skimmanine, dictamnine, dubinine, dubinidine, gerphytine, dubamine, and *N*-methylhaplofoline	Methanol	CC, IR, UV, NMR, MS, and X-ray	Whole plant	Uzbekistan	[73,74]
	Dubamine, dubinine, dubinidine, dictamnine, skimmianine, *N*-methylhaplofoline, gerphytine, and griffithine		CC, IR, UV, NMR, and MS	Aerial parts	Uzbekistan	[75]
	Flindersine, folimine, and evoxine		MP, TLC, UV, IR, NMR, and MS	Epigeal parts	Uzbekistan	[76]
*H. kowalenskyi* Stschegl.	Skimmianine and γ-fagarine		CC and TLC	Epigeal parts	Azerbaijan	[77]
*H. latifolium* Kar. & Kir.	Skimmianine, evoxine haplopine, glycoperine, 7-isopentenyloxy-γ-fagarine, haplamine, haplamide, haplamidine, and haplatine		CC, UV, IR, NMR, and MS	Whole plant	Kazakhstan	[78,79]
	Skimmianine, haplopine, haplamine, and POCS		HPLC-UV	Leaves	Uzbekistan	[53]
*H. leptomerum* Lincz. & Vved.	Isorhamnetin and haploside D	Ethanol	CC, MP, UV, NMR, and MS	Epigeal parts	Tajikistan	[68]
	β-Sitosterol, γ-fagarine, skimmianine, *N*-methyl-2-phenyl-4-quinolone, and leptomerine		MP, CC, UV, and NMR		Tajikistan	[80]
	Skimmianine, γ-fagarine, *N*-methyl-2-phenyl-4-quinolone, acutine, leptomerine, 2-heptylquinolin-4-one, and dictamnine	Methanol	CC, TLC, and NMR	Aerial parts	Tajikistan	[81]
	γ-Fagarine and dictamnine		CC, TLC, and NMR	Roots	Tajikistan	[81]
*H. multicaule* Vved.	β-Sitosterol, seselin and xanthyletin	Ethanol	CC, TLC, IR, NMR, and MP	Whole plant	Kazakhstan	[67]
*H. myrtifolium* Boiss.	Dictamnine, robustine, γ-fagarine, skimmianine, (-)-1β-polygamain, 7-*O*-(3-methyl-2-butenyl)-isodaurinol, and chrysosplenetin		CC, PTLC, UV, NMR, and MS	Aerial parts	Turkey	[82]
	Haplomyrtin and (−)-haplomyrfolin		CC, TLC, UV, NMR, and MS	Whole plant	Turkey	[83]
*H. pedicellatum* Bunge ex Boiss.	Scopoletin, 6-methoxymarmin, 7-geranyloxy-6-methoxycoumarin, and pedicellone	N.D.	TLC, CC, α_D_, IR, UV, and NMR	N.D.	N.D.	[84]
	γ-Fagarine, skimmianine, haplopine, haplamine, and POCS	Methanol	HPLC-UV	Leaves	Uzbekistan	[53]
	Skimmianine, γ-fagarine, haplopine, and robustine		CC, IR, UV, and NMR	Epigeal parts	Uzbekistan	[52]
	Haploside A, haploside B, and haploside C	Ethanol	CC, UV, NMR, and MS	Ground parts	Turkmenistan	[71]
	ECNP	N.D.	TFC methods	Aerial parts	Iran	[72]
*H. perforatum* Kar. & Kir.	Evoxine, haplopine, haplamine, skimmianine, and haplosamine	Methanol	CC, IR. UV, NMR, and MS	Epigeal parts	Kazakhstan	[85]
	Perforine, skimmianine, haplamine, haplopine, bucharaine, haplophyllidine, flindersine, and γ-fagarine		HPLC-UV	Leaves	Uzbekistan	[53]
	Evoxine, skimmianine, haplophyllidine, anhydroperlorine, flindersine, haplamine, and acetyl-haplophyllidine		CC, IR, UV, NMR, and MS	Aerial parts	Uzbekistan	[86]
	skimmianine, evoxine, 7-isopentenyloxy-γ-fagarine, perfamine, flindersine, haplamine, and eudesmin		CC, UV, MP, NMR, and MS	Epigeal parts	Uzbekistan	[87]
	Haplosinine, glycoperine, glucohaplopine, skimmianine, evoxine, haplamine, and 7-isopentenyloxy-γ-fagarine		CC, MP, NMR, and MS		Romania	[88,89]
	7-Isopentyloxy-γ-fagarine, skimmianine, evoxine, methylevoxine, glycoperine, haplamine, and flindersine		CC, UV, IR, NMR, and MS	Seeds and roots	Tajikistan	[90]
	Diphyllin		CC, IR, UV, NMR, and MS	Epigeal parts	Tajikistan	[49]
	Scopoletin, scopoletin 7-*O*-β-D-glucopyranoside, and haploperoside A	Ethanol	CC, UV, α_D_, IR, NMR, and MS		Kazakhstan	[91]
	Haploperoside B	Butanol	CC, UV, α_D_, IR, NMR, and MS		Kazakhstan	[91]
	Haploside A, haploside C, and haploside D	Ethanol	CC, α_D_, UV, IR, NMR, and MS		Kazakhstan	[92,93]
	Haploside E, haplogenin, and limocitrin-7-*O*-β-D-(6″-*O*-acetyl)-glucoside		CC, α_D_, UV, IR, NMR, and MS		Kazakhstan	[94]
*H. ptilosyylum* Spach	Justicin B, isodaurinol, matairesinol, arctigenin, (-)1β-polygamain, 4-[6″,7″-dihydroxygeranoyl]-matairesinol, 4-isopentylhaplomyrfolin A, 4-isopentylhaplomyrfolin B, picropolygamain, ptilostin, ptilostol, and ptilin	Methanol	CC, α_D_, UV, NMR, and MS	Aerial parts	Turkey	[95,96,97]
*H. ramosissimum* (Paulsen) Vved.	Skimmianine, haplopine, Haplamine, and γ-fagarine		HPLC-UV	Leaves	Uzbekistan	[53]
	Skimmianine, dictamnine, evoxine, scopoletin, and scoparone		CC, MP, IR, UV, NMR, and MS	Epigeal parts	Kazakhstan	[98]
*H. robustum* Bunge	ECNP	N.D.	Preliminary qualitative methods	Aerial parts	Iran	[72]
*H. schelkovnikovii* Grossh.	β-Sitosterol, obtusifol, and POCS	Chloroform and methanol	TLC, NMR, and IR	Epigeal parts	Azerbaijan	[99]
	Skimmianine and γ-fagarine	Methanol	CC and TLC		Azerbaijan	[77]
*H. sieversii* Fisch.	Flindersine, haplamine, anhydroevoxine, and eudesmin	Petroleum ether	CC, TLC, HPLC-UV, NMR, and MS	Aerial parts	Kazakhstan	[100]
*H. suaveolens* Ledeb.	Flindersine, γ-fagarine, kokusaginine, and haplophyllidine	Chloroform and benzene	CC, IR, UV, NMR, and MS	Whole plant	Turkey	[95]
	ECNP	Methanol and ethanol	Phytochemical screening	Aerial parts	Serbia	[50]
*H. tenue* Boiss.	Skimmianine and γ-fagarine	Methanol	CC and TLC	Epigeal parts	Azerbaijan	[77]
*H. telephioides* Boiss.	7-Hydroxy-9-methoxy-flindersine, diphyllin, 4-acetyl-diphyllin, and haplomyrtin	Ethanol	CC, UV, IR, NMR, and MS	Whole plant	Turkey	[96]
*H. thesioides* (Fisch. ex DC.) G.Don	Flindersine, kokusaginine, skimmianine, pteleine, nkolbisine, haplopline, haplosine, thesiolen, seselin, scoparone, and angustifolin	Chloroform	CC, IR, UV, NMR, and MS	Aerial parts	Turkey	[97]
*H. tuberculatum* Juss.	γ-Fagarine, skimmianine, and evoxine	Hot ethanol	CC, TLC, IR, UV, NMR, and MS		Iraq	[101]
	Flindersine and 3-dimethylallyl- 4-dimethylallyloxy-2-quinolone	*n*-Hexane	CC, IR, UV, NMR, and MS	Leaves and stems	Palestine	[102]
	(+)-Dihydroperfamine, 3-dimethylallyl-4-dimethylallyloxy-2-quinolone, tubasenecine, tubacetine, 7-hydroxy-8-(3-methyl-2-butenyl)-4-methoxyfuro2,3*b*-quinoline, justicidin A, and justicidin B	Dichloromethane	CC, TLC, UV, IR, NMR, and MS	Aerial parts	Saudi Arabia	[103]
	Tuberine	Petroleum ether and chloroform	CC, IR, UV, NMR, and MS		Lybia	[104,105]
	Skirnmianine, justicidin A, and diphyllin	Chloroform	CC, IR, UV, NMR, and MS		Sudan	[106]
	ECNP	N.D.	Preliminary qualitative methods		Iran	[72]
	Haplotubinone, haplotubine, dyphyllin, and *N*-(2-phenylethyl)-benzamide	Dichloromethane	CC, IR, UV, NMR, MS, and X-ray		Saudi Arabia	[107]
	Skimmianine and γ-fagarine	Petroleum ether	CC, TLC, NMR, and MS		Iraq	[108]
	Ammoidin and POCS		TLC, MP, and HPLC-UV			
	1-Hydroxy-3-(hydroxymethyl)-6,7-dimethoxy-4-(3,4-methylenedioxyphenyl)- 2-naphthoic acid γ-lactone, and (−)-secoisolariciresinol	Methanol	CC, IR, HPLC-UV, NMR, and MS	Whole plant	Egypt	[109]
	5,7,4′-Trihydroxy-6-methoxy-3-*O*-glucosyl flavone	Ethyl acetate	CC, IR, UV, NMR, and MS	Aerial parts	Sudan	[106]
	justicidin A, justicidin B, tuberculatin, and acetyl-tuberculatin	Methanol	CC, TLC, NMR, and HPLC-DAD	Aerial parts	Spain	[110]
*H. vulcanicum* Boiss. & Heldr.	Vulcanine, dictamnine, γ-fagarine, robustine, haplopine, skimmianine, nigdenine, scopoletin, umbelliferone, (−)-haplomyrfolin, kusunokinin, diphyllin, syringarasinol, tuberculatin, haplomyrfolol, and konyanin	Ethanol	CC, IR, UV, NMR, and MS	Whole plant	Turkey	[111,112,113]

α_D_: Optical Rotation; CC: Column Chromatography; ECNP: Exact Compounds Not Specified; HPLC-DAD: High Performance Liquid Chromatography Coupled to Diode Array Detector; HPLC-PDA-MS: High Performance Liquid Chromatography Coupled to Photodiode Array Detector and Mass spectrometry; HPLC-UV: High Performance Liquid Chromatography Coupled to Ultraviolet Spectroscopy; IR: Infrared Spectroscopy; MP: Melting Point; MS: Mass Spectrometry; N.D.: Not Reported; NMR: Nuclear Magnetic Resonance spectroscopy; POCS: Plus Other Compounds Not Specified; PTLC: Preparative Thin Layer Chromatography; SPE-NMR: Solid Phase Extraction with Nuclear Magnetic Resonance Spectroscopy; TLC: Thin Layer Chromatography; UV: Ultraviolet Spectroscopy; X-ray: X-Ray Spectroscopy.

**Table 4:** Distribution of the non-volatile phytochemicals in the *Haplophyllum* genus.

Phytochemical Class	Phytochemical Compound	*Haplophyllum* spp.	References
Alkaloids	2-Heptylquinolin-4-one	*H. leptomerum*	[81]
	2-Nonyl-quinolin-4(1*H*)-one	*H. acutifolium*	[43]
	3-Dimethylallyl-4-dimethylallyloxy-2-quinoline	*H. bucharicum* *H. tuberculatum*	[49,102,103]
	4-Hydroxyquinolin-2-one	*H. bucharicum*	[51]
	4-Methoxyquinolin-2-one	*H. bucharicum* *H. bungei*	[51,54]
	4-Methoxy-*N*-methyl-2-quinolone	*H. dauricum*	[66]
	7-Hydroxy-9-methoxy-flindersine	*H. telephioides*	[96]
	7-Hydroxy-8-(3-methyl-2-butenyl)-4-methoxyfuro2,3*b*-quinoline	*H. tuberculatum*	[103]
	7-Isopentenyloxy-γ-fagarine	*H. canaliculatum* *H. latifolium* *H. perforatum*	[57,78,87,89,90]
	γ-Fagarine	*H. bucharicum* *H. bungei* *H. dauricum* *H. kowalenskyi* *H. leptomerum* *H. myrtifolium* *H. pedicellatum* *H. perforatum* *H. ramosissimum* *H. schelkovnikovii* *H. suaveolens* *H. tenue* *H. tuberculatum* *H. vulcanicum*	[51,52,53,66,77,80,81,82,95,101,108,111]
	*N*-methyl-2-phenyl-4-quinolone	*H. foliosum* *H. leptomerum*	[43,80,81]
	*N*-methylhaplofoline	*H. griffithianum*	[73,75]
	(+)-Dihydroperfamine	*H. tuberculatum*	[103]
	Acutine	*H. acutifolium* *H. leptomerum*	[43,81]
	Anhydroevoxine	*H. sieversii*	[100]
	Anhydroperlorine	*H. perforatum* *H. sieversii*	[86]
	Acetyl-haplophyllidine	*H. perforatum*	[86]
	Atanine	*H. canaliculatum*	[57]
	Bucharaine	*H. bucharicum* *H. perforatum*	[49,51,52,53]
	Bucharaminol	*H. bucharicum*	[51]
	Bucharidine	*H. bucharicum*	[51,52]
	Daurine	*H. dauricum*	[66]
	Dictamnine	*H. bucharicum* *H. bungei* *H. cappadocicum* *H. dauricum* *H. griffithianum* *H. leptomerum* *H. myrtifolium* *H. ramosissimum* *H. vulcanicum*	[51,53,54,58,66,73,75,81,82,98,111]
	Dubamine	*H. griffithianum*	[73,75]
	Dubinine	*H. griffithianum*	[73,75]
	Dubinidine	*H. foliosum* *H. griffithianum*	[70,73,75]
	Edulinine	*H. foliosum*	[70]
	Evoxine	*H. acutifolium* *H. griffithianum* *H. latifolium* *H. perforatum* *H. ramosissimum* *H. tuberculatum*	[45,76,78,85,86,87,89,90,98,101]
	Flindersine	*H. acutifolium* *H. bucharicum* *H. canaliculatum* *H. griffithianum* *H. perforatum* *H. sieversii* *H. suaveolens* *H. thesioides* *H. tuberculatum*	[47,51,53,57,75,86,87,90,95,97,100,102]
	Folidine	*H. foliosum*	[70]
	Folifine	*H. bucharicum*	[52]
	Folimine	*H. bungei* *H. dauricum* *H. foliosum* *H. griffithianum*	[46,54,66,69,76]
	Foliosidine	*H. foliosum*	[46,69]
	Foliosine	*H. foliosum*	[46,70]
	Gerphytine	*H. griffithianum*	[74,75]
	Glucohaplopine	*H. perforatum*	[89,90]
	Glycoperine	*H. perforatum*	[89]
	Griffithine	*H. griffithianum*	[75]
	Haplacutine A	*H. acutifolium*	[43,44]
	Haplacutine B	*H. acutifolium*	[43]
	Haplacutine C	*H. acutifolium*	[43]
	Haplacutine D	*H. acutifolium*	[43]
	Haplacutine E	*H. acutifolium*	[43]
	Haplacutine F	*H. acutifolium*	[43]
	Haplamide	*H. latifolium*	[78]
	Haplamidine	*H. latifolium*	[78]
	Haplamine	*H. acutifolium* *H. bucharicum* *H. bungei* *H. latifolium* *H. pedicellatum* *H. perforatum* *H. ramosissimum* *H. sieversii*	[43,51,53,78,85,86,87,89,90,100]
	Haplatine	*H. latifolium*	[79]
	Haplobungine	*H. bungei*	[54]
	Haplodimerine	*H. foliosum*	[46]
	Haplophyllidine	*H. perforatum* *H. suaveolens*	[53,86,95]
	Haplopine	*H. bucharicum* *H. bungei* *H. cappadocicum* *H. dauricum* *H. latifolium* *H. pedicellatum* *H. perforatum* *H. ramosissimum* *H. thesioides* *H. vulcanicum*	[51,52,53,58,66,78,85,97,111]
	Haplosamine	*H. perforatum*	[85]
	Haplosinine	*H. perforatum* *H. thesioides*	[88,97]
	Haplotin	*H. acutifolium*	[46]
	Haplotubine	*H. tuberculatum*	[107]
	Haplotubinone	*H. tuberculatum*	[107]
	Haplophytin-A	*H. acutifolium*	[47]
	Haplophytin-B	*H. acutifolium*	[47]
	Kokusaginine	*H. suaveolens* *H. thesioides*	[95,97]
	Leptomerine	*H. leptomerum*	[80,81]
	Malatyamine	*H. cappadocicum*	[61]
	Methylevoxine	*H. perforatum*	[90]
	Nigdenine	*H. vulcanicum*	[111]
	Nkolbisine	*H. thesioides*	[97]
	Perfamine	*H. canaliculatum* *H. perforatum*	[57,87]
	Perforine	*H. perforatum*	[53]
	Pteleine	*H. thesioides*	[97]
	Robustine	*H. bucharicum* *H. cappadocicum* *H. dauricum* *H. myrtifolium* *H. pedicellatum* *H. vulcanicum*	[51,52,58,66,82,111]
	Robustinine	*H. bungei* *H. dauricum*	[54,62]
	Skimmianine	*H. acutifolium* *H. bucharicum* *H. bungei* *H. canaliculatum* *H. cappadocicum* *H. dauricum* *H. foliosum* *H. griffithianum* *H. kowalenskyi* *H. latifolium* *H. leptomerum* *H. myrtifolium* *H. pedicellatum* *H. perforatum* *H. ramosissimum* *H. schelkovnikovii* *H. tenue* *H. thesioides* *H. tuberculatum* *H. vulcanicum*	[44,45,46,49,51,52,53,54,57,58,66,73,74,77,78,80,81,82,85,86,87,89,90,97,98,101,106,108,111]
	Tubacetine	*H. tuberculatum*	[103]
	Tubasenecine	*H. tuberculatum*	[103]
	Tuberine	*H. tuberculatum*	[104,105]
	Vulcanine	*H. vulcanicum*	[112]
Coumarins	5,7-Dihydroxy-coumarin	*H. dauricum*	[62]
	5-Hydroxy-7-methoxycoumarin	*H. bungei*	[55]
	6-Methoxymarmin	*H. pedicellatum*	[84]
	7-(3′,3′-Dimethylallyloxy)-6-methoxycoumarin	*H. bungei*	[55]
	7-Geranyloxy-6-methoxycoumarin	*H. pedicellatum*	[84]
	Ammoidin	*H. tuberculatum*	[108]
	Angustifolin	*H. thesioides*	[97]
	Bungeidiol	*H. bungei*	[56]
	Dauroside A	*H. dauricum*	[63,64]
	Dauroside B	*H. dauricum*	[63,64]
	Dauroside C	*H. dauricum*	[65]
	Dauroside D	*H. dauricum*	[60]
	Haploperoside A	*H. perforatum*	[91]
	Haploperoside B	*H. perforatum*	[91]
	Isoscopoletin	*H. bungei*	[56]
	Obtusifol	*H. schelkovnikovii*	[99]
	Osthole	*H. bungei*	[55]
	Pedicellone	*H. pedicellatum*	[84]
	Ptilin	*H. ptilosyylum*	[96,97]
	Ptilostin	*H. ptilosyylum*	[96,97]
	Ptilostol	*H. ptilosyylum*	[96,97]
	Scoparone	*H. ramosissimum* *H. thesioides*	[97,98]
	Scopoletin	*H. bungei* *H. cappadocicum* *H. dauricum* *H. dubium* *H. pedicellatum* *H. perforatum* *H. ramosissimum* *H. vulcanicum*	[56,58,62,68,84,91,98,111]
	Scopoletin 7-*O*-β-D-glucopyranoside	*H. perforatum*	[91]
	Scopolin	*H. dubium*	[68]
	Seselin	*H. cappadocicum* *H. dshungaricum* *H. multicaule* *H. thesioides*	[58,67,97]
	Yhesiolen	*H. thesioides*	[97]
	Umbelliferone	*H. dauricum* *H. vulcanicum*	[62,111]
	Umbelliferone 7-*O*-β-D-glucoside	*H. dauricum*	[62]
	Xanthyletin	*H. dshungaricum* *H. multicaule*	[67]
Flavonoids	5,7,4′-Trihydroxy-6-methoxy-3-*O*-glucosyl flavone	*H. tuberculatum*	[106]
	Chrysosplenetin	*H. myrtifolium*	[82]
	Haplogenin	*H. perforatum*	[94]
	Haploside A	*H. pedicellatum* *H. perforatum*	[71,102]
	Haploside B	*H. dauricum* *H. dubium* *H. pedicellatum*	[65,68,71]
	Haploside C	*H. foliosum* *H. pedicellatum* *H. perforatum*	[71,93]
	Haploside D	*H. dauricum* *H. dubium* *H. leptomerum* *H. perforatum*	[65,68,93]
	Haploside E	*H. perforatum*	[94]
	Isorhamnetin	*H. foliosum* *H. leptomerum*	[68,71]
	Limocitrin-7-*O*-β-D-(6″-*O* acetyl)-glucoside	*H. foliosum* *H. perforatum*	[71,94]
Lignans	1-Hydroxy-3-(hydroxymethyl)-6,7-dimethoxy-4-(3,4-methylenedioxyphenyl)-2-naphthoic acid γ-lactone	*H. tuberculatum*	[109]
	4-[6″,7″-Dihydroxygeranoyl]-matairesinol	*H. ptilosyylum*	[95]
	4-Acetyl-diphyllin	*H. bucharicum* *H. telephioides*	[51,96]

	4-Isopentylhaplomyrfolin A	*H. ptilosyylum*	[95,96]
	4-Isopentylhaplomyrfolin B	*H. ptilosyylum*	[95,96]
	7-*O*-(3-Methyl-2-butenyl)-isodaurinol	*H. myrtifolium*	[82]
	(−)-lβ-Polygamain	*H. cappadocicum* *H. myrtifolium* *H. ptilosyylum*	[60,82,95,96]
	(−)-Cappodicin	*H. cappadocicum*	[59]
	(−)-Cappadoside	*H. cappadocicum*	[59]
	(−)-Haplodoside	*H. cappadocicum*	[59]
	(−)-Haplomyrfolin	*H. myrtifolium* *H. vulcanicum*	[83,111]
	(−)-Haplomyrtoside	*H. cappadocicum*	[60]
	(−)-Majidine	*H. cappadocicum*	[60]
	(−)-Secoisolariciresinol	*H. tuberculatum*	[109]
	Acetyl-tuberculatin	*H. tuberculatum*	[110]
	Arctigenin	*H. ptilosyylum*	[95,96]
	Daurinol	*H. cappadocicum* *H. dauricum*	[58,62]
	Diphyllin	*H. alberti-regelii* *H. bucharicum* *H. cappadocicum* *H. dauricum* *H. perforatum* *H. telephioides* *H. tuberculatum* *H. vulcanicum*	[49,51,58,65,96,106,111]
	Eudesmin	*H. acutifolium* *H. perforatum* *H. sieversii*	[46,48,87,100]
	Haplomyrtin	*H. myrtifolium* *H. telephioides*	[82,96]
	Haplomyrfolol	*H. vulcanicum*	[111]
	Isodaurinol	*H. cappadocicum* *H. ptilosyylum*	[58,95,96]
	Justicidin A	*H. cappadocicum* *H. tuberculatum*	[58,103,106,110]
	Justicidin B	*H. bucharicum* *H. cappadocicum* *H. dauricum* *H. ptilosyylum* *H. tuberculatum*	[51,58,62,95,96,103,110]
	Konyanin	*H. vulcanicum*	[112]
	Kusunokinin	*H. acutifolium* *H. vulcanicum*	[47,111]
	Matairesinol	*H. cappadocicum* *H. ptilosyylum*	[58,95,96]
	Picropolygamain	*H. ptilosyylum*	[95,96]
	Syringarasinol	*H. vulcanicum*	[111]
	Tuberculatin	*H. tuberculatum*	[110]
Others	*N*-(2-Phenylethyl)-benzamide	*H. tuberculatum*	[107]
	Acetamide	*H. acutifolium*	[44]
	Benzamide	*H. bucharicum*	[52]
	Ferulic acid	*H. foliosum*	[70]
	Vanillic acid	*H. cappadocicum*	[60]
Terpenoids	β-Sitosterol	*H. acutifolium* *H. bucharicum* *H. leptomerum* *H. multicaule* *H. schelkovnikovii*	[47,49,67,80,99]
	Campesterol	*H. bucharicum*	[49]
	Cholesterol	*H. acutifolium* *H. bucharicum*	[47,49]
	Oleanolic acid	*H. acutifolium*	[47]
	Stigmasterol	*H. bucharicum*	[49]
